# Mapping and Characterization of the Interaction Interface between Two Polypyrimidine-Tract Binding Proteins and a Nova-Type Protein of *Solanum tuberosum*

**DOI:** 10.1371/journal.pone.0064783

**Published:** 2013-05-24

**Authors:** Shweta Shah, Nathaniel M. Butler, David J. Hannapel, A. Gururaj Rao

**Affiliations:** 1 Roy J. Carver Department of Biochemistry Biophysics and Molecular Biology, Iowa State University, Ames, Iowa, United States of America; 2 Plant Biology Major, Iowa State University, Ames, Iowa, United States of America; Institute of Enzymology of the Hungarian Academy of Science, Hungary

## Abstract

Polypyrimidine tract-binding (PTB) proteins are RNA-binding proteins that generally contain four RNA recognition motifs (RRMs). In potato, six cDNAs encoding full-length PTB proteins have been identified. In the present study Nova1-like protein, designated *St*Nova1, was identified as a potential interacting partner of the *St*PTB proteins via yeast two-hybrid screening. Nova protein is a RNA-binding protein that contains three K-homology (KH) domains. In humans, these proteins are involved in regulation of neuronal RNA metabolism but the role of Nova-like proteins in plants is poorly understood. We have validated this interaction and mapped the protein binding region on *St*Nova1 and *St*PTB1 and −6 using a novel domain interaction phage display (DIPP) technique. The interaction between the two RNA-binding proteins *St*PTB1/6 and *St*Nova1 is mediated through linker regions that are distinctly separated from the RRMs. Furthermore, using a random 21-mer phage-peptide library, we have identified a number of peptides with the consensus sequence motif [S/G][V/I][L/V]G that recognize the *St*PTB proteins. One over-represented peptide that recognizes StPTB6 contains the GVLGPWP sequence that is similar to the GIGGRYP sequence in the glycine-rich linker region between the KH2 and KH3 domains of *St*Nova1. We show, through site-specific mutations, the importance of glycine and proline residues in *St*Nova1-*St*PTB interactions.

## Introduction

Polypyrimidine tract-binding (PTB) proteins are RNA-binding proteins that have been well characterized and studied in vertebrates. They typically contain four RNA recognition motifs (RRM) that are separated by three linker regions and all the RRM domains have been shown to be involved in RNA binding. The RRMs interact with sequence in UTRs containing groups of four cytosine/uracil motifs at least four nucleotides in length [1], [2].The PTBs participate in multiple regulatory functions in mRNA metabolism including polyadenylation, 3′ end formation, translation from internal ribosomal entry sites, RNA localization and stability, and alternative splicing (AS) [2], [3], [4]. In some instances, the function of PTB proteins is mediated via primary interactions with other regulatory proteins. Thus, PTB proteins have been reported to interact with proteins such as Raver, Nova-1, Nova-2, and MRG15 proteins [5], [6], [7], [8], [9], [10]. Raver1 is a protein that promotes AS of α-tropomyosin and consists of three RRM domains [11]. The Raver1 protein has an extended C-terminal region with four conserved PTB interaction motifs containing the consensus sequence [S/G][I/L]LGxxP [8], [11]. The Nova (Neuron Oncological Ventral Antigen) proteins are neuron specific RNA binding proteins containing three RNA-binding domains, referred to as K-homology or KH domains. The first and second domains are in tandem arrangement, followed by a large spacer region and the third domain at the C-terminal of the protein. These proteins are also involved in controlling AS [5]. The Nova1-like protein of *Arabidopsis thaliana*, designated BTR1 (binding to ToMV RNA 1L), binds specifically to terminal regions of genomic RNA of the tomato mosaic virus (ToMV) [12]. These terminal regions contain important regulatory elements for translation and RNA replication. In planta analysis suggests that by binding to the viral RNA, BTR1 regulates replication and cell-to-cell movement of ToMV [12]. The Nova-1 family of genes is highly conserved within the plant kingdom with orthologs in numerous diverse species, including *Glycine max*, *Hordeum vulgare*, *Medicago truncatula*, *Oryza sativa*, *Populus trichocarpa*, *Ricinus communis*, *Solanum lycopersicum*, *Vitis vinifera*, and *Zea mays*. Despite this conservation, however, with the exception of BTR1, very little is known about the function of these plant orthologs.

PTB proteins and other RNA-binding proteins involved in diverse aspects of RNA metabolism also occur in plants [13], [14]. In *Arabidopsis thaliana* there are three PTB-homologs (*At*PTB1−3). *At*PTB1 and −2 contain three RRMs, whereas *At*PTB3 contains four. Using a transcriptome-wide analysis, AS activity was observed for *At*PTB1 and −2 but no activity was detected for the distantly related *At*PTB3 [15]. Importantly, their localization to distinct cellular compartments (nucleus, cytosol and processing bodies) attest to their multifunctional role in developmental processes [16]. PTB-related proteins in *Arabidopsis* have also been implicated in pollen germination possibly through their function in processes of RNA metabolism [17]. In pumpkin, RBP50, a phloem-mobile PTB protein, similar to *At*PTB3, functions as a non-cell-autonomous RNA-binding protein, is highly enriched in the phloem sap and forms the core of a large ribonucleoprotein complex containing at least sixteen proteins and six RNAs [13].

Recently in potato (*Solanum tuberosum*), six cDNAs that encode full-length PTB proteins have been identified [18]. Of these, two are *At*PTB3-types containing four RRMs (*St*PTB1 and *St*PTB6) and four are 3-RRM types (*St*PTB7, TC201749, B1920231 and TC218925). It has been demonstrated that the *St*PTB1 and −6 genes have distinct expression patterns and respond to both developmental and environmental cues [18]. To elucidate the function of these two RBP50-like proteins in potato and identify putative protein partners that may facilitate their binding to phloem RNAs, we have focused our attention on protein-protein interactions mediated by *St*PTB1 and −6. In the present work, using *St*PTB1 protein as bait in yeast two-hybrid experiments, we have identified a Nova-1 like protein (*St*Nova1) and characterized the interaction between the two PTB proteins *St*PTB1 and −6, and *St*Nova1 using multiple techniques. We also describe the use of a modified phage display technique, termed **D**omain **I**nteraction **P**hage **P**anning (DIPP), to identify the binding site interface between the two proteins and provide molecular insights for further mutagenesis experiments.

## Materials and Methods

### Yeast Two-hybrid Screening

The yeast two-hybrid screening was performed as described previously [19]. Briefly, the full length *St*PTB1 was cloned into the pBridge vector (CLONTECH) and used as bait to screen ∼10^6^ transformants from a leaf cDNA library of potato (*Solanum tuberosum* cv Désirée) in pAD-GAL4-2.1 (Stratagene, La Jolla, CA). Positive interactions were confirmed by co-transforming yeast strain AH109 with each purified pAD plasmid and pBridge: *St*PTB1 and plating on -Leu/−Trp (transformation control).

### Plasmid Construction

The *St*PTB1 and *St*PTB6 were cloned into pE-SUMO vector (LifeSensors) using the primers as mentioned in Table S1. The forward primer incorporated BsaI site and the reverse primer incorporated BsaI and XbaI sites. The PCR products were ligated into pE-SUMO vector that adds N-terminus SUMO and 6xH tag to PTB proteins. The *St*Nova1 was cloned into modified pGEX4T-1-TEV vector for ligation independent cloning using the primer mentioned in Table S1. For cloning *St*Nova1 domain (D5), region between 571−780 was amplified by PCR using *St*Nova1 cDNA as a template. The forward primer incorporated SfiI site and reverse primer has NotI site (see Table S1). The PCR fragments were digested with SfiI and NotI and ligated into similarly digested pGEX4T-1. Sub-regions (designated as S1, S2 and S3) of domain D5 were also similarly cloned into pGEX4T-1 vector.

### Protein Expression and Purification

Proteins were expressed overnight in Rosetta2 (DE3) pLysS (Novagen) cells at 20°C with 0.5 mM IPTG induction. For purification of Glutathione S-transferase (GST) fused protein, cell pellets were suspended in buffer A (1×Tris-buffer saline pH 7.4, 0.1% Triton X-100, 1 mM DTT and 1 mM PMSF) and lysed by sonication on ice. The lysate was centrifuged at 13,000 rpm for 20 min and clarified supernatant was loaded onto glutathione agarose (Pierce) resins that were already equilibrated with buffer A. The unbound protein was washed with buffer B (50 mM Tris-HCl pH-8 and 1 mM DTT) and elution was done with buffer B containing 10 mM glutathione. For 6xH tagged proteins, the cell pellets were lysed into buffer C (50 mM Tris-HCl pH 8.0, 40 mM imidazole, 0.1% triton X-100, 1 mM DTT and 1 mM PMSF) and the protein was purified using Ni-NTA superflow resins. After washing the unbound proteins with buffer B containing 40 mM imidazole, the protein was eluted with Buffer B containing 150 mM imidazole. Protein concentrations were determined by the Bradford method [20].

### Pull Down Assays

The MBP-*St*Nova1 protein was used as bait to pull out SUMO-*St*PTB proteins. Purified MBP-*St*Nova1 (20 µg) was bound on amylose resin (20 µl) and washed three times with wash/binding buffer (50 mM Tris-HCl pH 7.4, 100 mM NaCl, 1 mM DTT, 0.1% NP-40). The resin-bound MBP-*St*Nova protein was subsequently added to a solution containing SUMO-*St*PTB protein (30 µg) in binding buffer in a final volume of 500 µl, followed by incubation with rotation for 1 h at room temperature. As a negative control MBP alone was incubated with SUMO-*St*PTB and SUMO proteins. After washing four times with wash/binding buffer to remove unbound protein, the resin was boiled at 95°C for 5 min in SDS-PAGE sample buffer, proteins resolved by 12% SDS-PAGE and analyzed by coomassie staining or by western blots using anti-SUMO primary antibody (Rockland Immunochemicals, 1∶2000) and polyclonal alkaline phosphate (AP) conjugated anti-rabbit secondary antibody (Sigma, 1∶20,000). Colorimetric detection of secondary antibody was performed using the AP substrate kit (Bio-Rad).

### Construction of Phage-displayed Overlapping Domains of *St*Nova1

Six overlapping domains of *St*Nova1 (D1–D6) comprising amino acid residues 1–76 (D1), 39–129 (D2), 62–129(D3), 110–205(D4), 190–260(D5), 246–326(D6) were generated by PCR using *St*Nova1 cDNA as a template and forward and reverse primers to incorporate SfiI and NotI sites (Table S1). The PCR fragments were then digested with SfiI and NotI, ligated into a similarly digested pCANTAB 5E phagemid vector and subsequently transformed into *E. Coli* XL1-Blue cells (Stratagene). Single colonies from each plate were inoculated into 5 ml 2YT/carb/Amp media, grown to OD at 600 λ∼0.2–0.3 at 37°C and then infected with helper phage-VCSM13 (Stratagene). After 1 hour the cell culture was transferred to 25 ml 2YT/Kan media and further incubated overnight at 37°C. After removing cell debris by centrifugation, phage particles were precipitated from the supernatant using 7.5 ml of 20% PEG solution containing NaCl. The precipitate was re-suspended in 1 ml of PBS and phage concentration determined by measuring absorbance at 268 λ (OD_268_ = 1.0 for a solution containing 5×10^12^ phage per ml). A “domain library” was prepared by mixing equal concentrations of individual phage-domains.

### Domain Interaction Phage Panning (DIPP)

SUMO fused PTB1, PTB6 and SUMO (control) proteins were immobilized in the wells of a nunc maxisorp ELISA plate by aliquoting 100 µl of each protein at a concentration of 10 µg/ml in 50 mM NaHCO_3_, pH 9.6, at room temperature with gentle rotation for 2 h. Wells were then blocked with PBS containing 0.2% BSA for 1 h followed by 3 washings with PBS containing 0.05% Tween 20 (PBST) and then incubated with 100 µl of domain phage library for 3 h at room temperature with gentle rotation. After removing unbound phage by washing 5 times with PBST, bound phage was eluted by incubating with 500 µl of 0.1 M HCl for 5 min at room temperature with shaking. The eluted phage was immediately neutralized by the addition of 1/3^rd^ phage volume of 1 M Tris-HCl buffer pH 8.0, followed by infection of XL1-Blue cells (grown to <0.6 OD) with the phage. Infected XL1-Blue cells were incubated at 37°C for 20 min followed by addition of the helper phage (VCSM13) and again incubating at 37°C for 30 min. The phage infected XL1-Blue cells were transferred into a conical flask containing 50 ml of 2YT media containing 10 µg/ml of tetracycline and 100 µg/ml ampicillin that was further incubated at 37°C overnight with shaking at 210 rpm. Phage preparation was carried out as described earlier. This process yielded the first round of enriched phage. The entire process was repeated for one more cycle and after the 2^nd^ round, phage infected XL1-Blue cells were grown on 2YT ampicillin plates. Plasmids were prepared from 20 randomly selected colonies and their DNA sequenced.

### Phage ELISA for Binding Specificity

For phage ELISAs, 100 µl of GST fused proteins and GST (control) (10 µg/ml in 50 mM NaHCO_3_, pH 9.6) were immobilized in the wells of an ELISA plate at room temperature with gentle rotation for 2 h. Plate was then washed two times with PBS followed by blocking with PBST containing 0.2% BSA for 1 h. Subsequently, after three washings with PBST, wells were incubated with the 100 µl of appropriate domain phage diluted in PBST containing 0.2% BSA for 2 h at room temperature with gentle shaking. The plate was again washed three times with PBST followed by incubation with anti-M13 HRP conjugated antibody for 1 h. After washing four times with PBST, bound phage in each well was detected by incubating with 50 µl of substrate solution (0.01% hydrogen peroxide +0.8 mg/ml o-Phenylenediamine dihydrochoride) for ∼10 min. Reactions were terminated by the addition of 50 µl of 3 M HCl and measuring the absorbance of the developed yellow color at 490 nm. Data were generated by subtracting the absorbance of the control (SUMO or GST) wells from the experimental wells.

### Protein Overlay Assays

For protein overlays assays, 2 µg of GST fused recombinant proteins were separated on 12% SDS-PAGE and blotted on to PVDF membrane. Blots were then blocked with 5% milk protein in Tris-buffered saline +0.1% Tween 20 (TBST) for 1 h. Blots were then washed three times with TBST (5 min) and incubated with the SUMO-fused protein or SUMO protein (for control) at a concentration of 25 nM for 1 h at room temperature. The binding of SUMO-fused proteins and SUMO proteins were detected by using anti-SUMO primary antibody as described in the section on pull down assays.

## Results

### Identification of PTB Interacting Proteins

**Figure 1:**
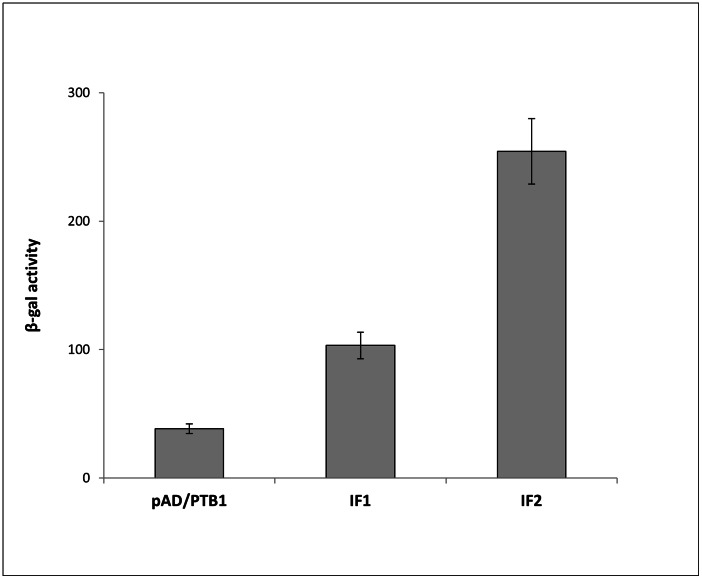
Yeast 2-hybrid interaction of *St*PTB1 with IF1 and IF2. Induction of β-galactosidase activity is used to assess the degree of interaction. Interaction of *St*PTB1 expressed in pBridge with an empty pAD vector is shown as a negative control. Both IF1 and IF2 show statistically significant differences in β-galactosidase activity compared to the PTB1+ pAD negative control.

**Figure 2:**
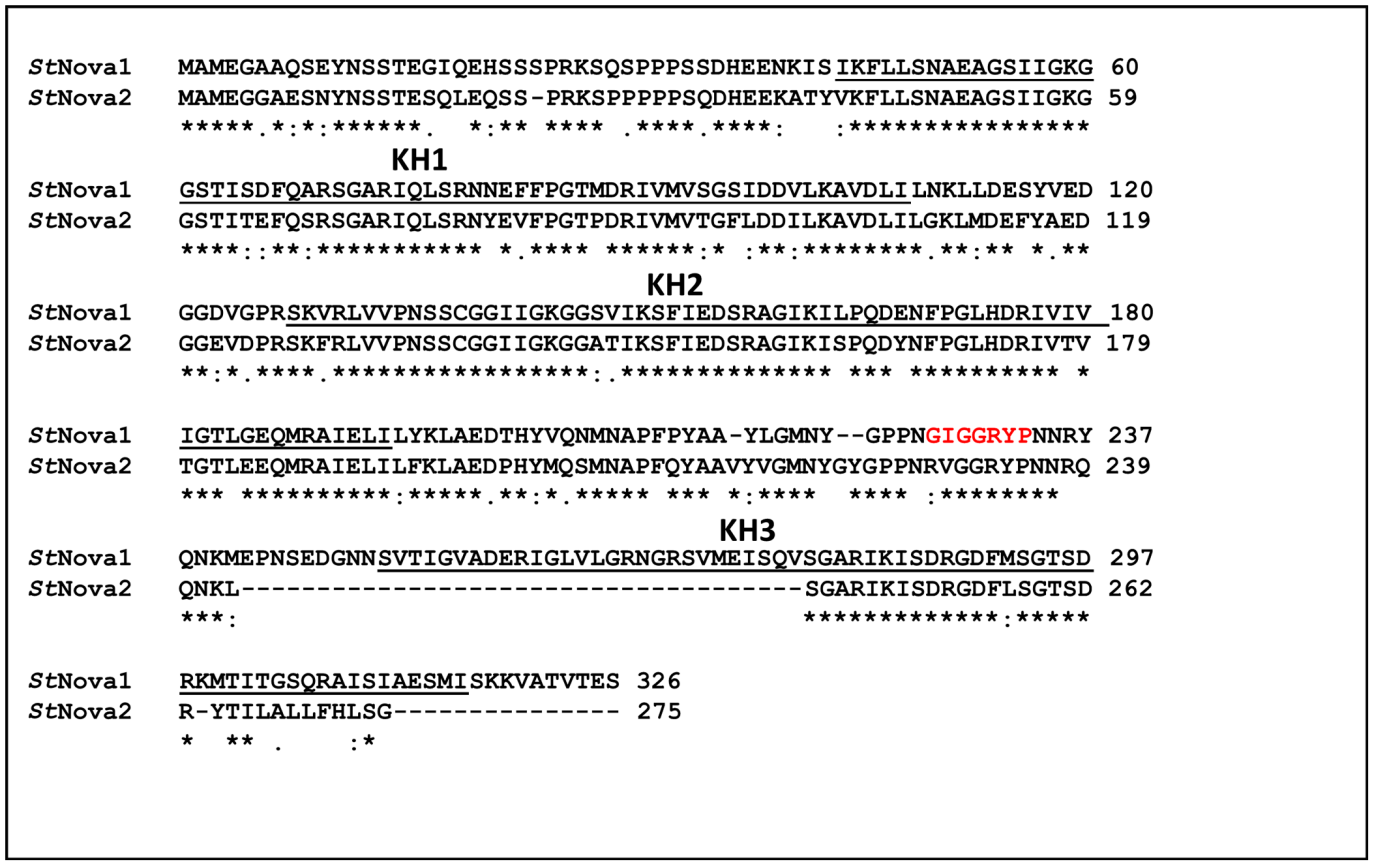
Amino acid sequence alignment of two isoforms of *St*Nova proteins. The KH domains are underlined. Note the missing portion of the third KH domain in *St*Nova2. The GxxGxxP motif present on *St*Nova1 is highlighted in red.

*St*PTB1 and *St*PTB6 are similar to CmRBP50 (>84% sequence identity among the proteins), the core protein in a phloem-mobile protein-RNA complex containing 16 proteins and six RNAs [13]. Therefore, it is reasonable to infer that the potato PTB proteins function similarly through protein-protein and protein-RNA interactions. To identify potential proteins interacting with *St*PTB proteins we performed a yeast two-hybrid screening against leaf proteins using *St*PTB1 as bait. Based on the induction of β-galactosidase activity (Fig. 1), two interacting clones, designated as IF1 and IF2, were identified. Sequence analysis revealed that IF2 exhibited a sequence match (22.8% identity/∼38% similarity) to the mammalian RNA-binding protein, Nova, containing three KH domains [5] and is therefore referred to as *St*Nova1. The second clone, IF1, was found to be a putative phosphonucleolar protein containing three RRMs but is not discussed further in this paper. We cloned the full-length 35.2 kDa *St*Nova1 from a potato cDNA library and also identified an isoform, *St*Nova2, which is missing a portion of the third KH domain (Fig. 2).

### Interaction between *St*PTB Protein and *St*Nova1 Protein Analyzed by Pull-down Assays

**Figure 3:**
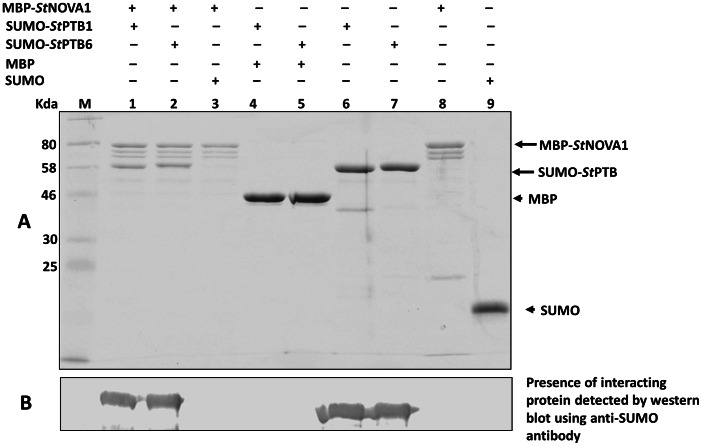
Interaction between *St*PTB and *St*Nova1 proteins demonstrated by pull-down assays. (**A**) Coomassie stained SDS PAGE showing interaction between MBP*-St*Nova1 protein with SUMO-*St*PTB1 (lane 1) and SUMO-*St*PTB6 (lane 2) while the controls MBP-*St*Nova1 and SUMO (lane 3) and MBP and *St*PTB proteins (lane 4 and 5) do not show any interaction. Lanes 6–9 shows the purified *St*PTB1, *St*PTB6, MBP-*St*Nova1 and SUMO proteins respectively used for interaction. (**B**) Western blot using anti-SUMO antibody demonstrating the presence of interacting protein in lanes 1, 2, 6 and 7.

In a parallel approach we assessed the interaction between *St*PTB and *St*Nova1 proteins by pulldown assays. For this purpose, *St*PTB proteins were expressed and purified as 6His-SUMO fusion and *St*Nova1 as MBP fusion. Both the SUMO-PTB proteins were pulled down by MBP-*St*Nova1 (Fig. 3A, lanes 1 and 2). The specificity of this interaction was confirmed by the absence of interaction of MBP-*St*Nova1 with SUMO protein alone (Fig. 3A, lane 3). In contrast, the MBP protein by itself was unable to pull-down the SUMO-PTB proteins (Fig. 3A, lanes 4 and 5). The presence of SUMO-PTB in lanes 1, 2, 6 and 7 was also confirmed with western blots using polyclonal, anti-SUMO antibody (Fig. 3B).

### Phage Panning of *St*PTB Proteins

**Figure 4:**
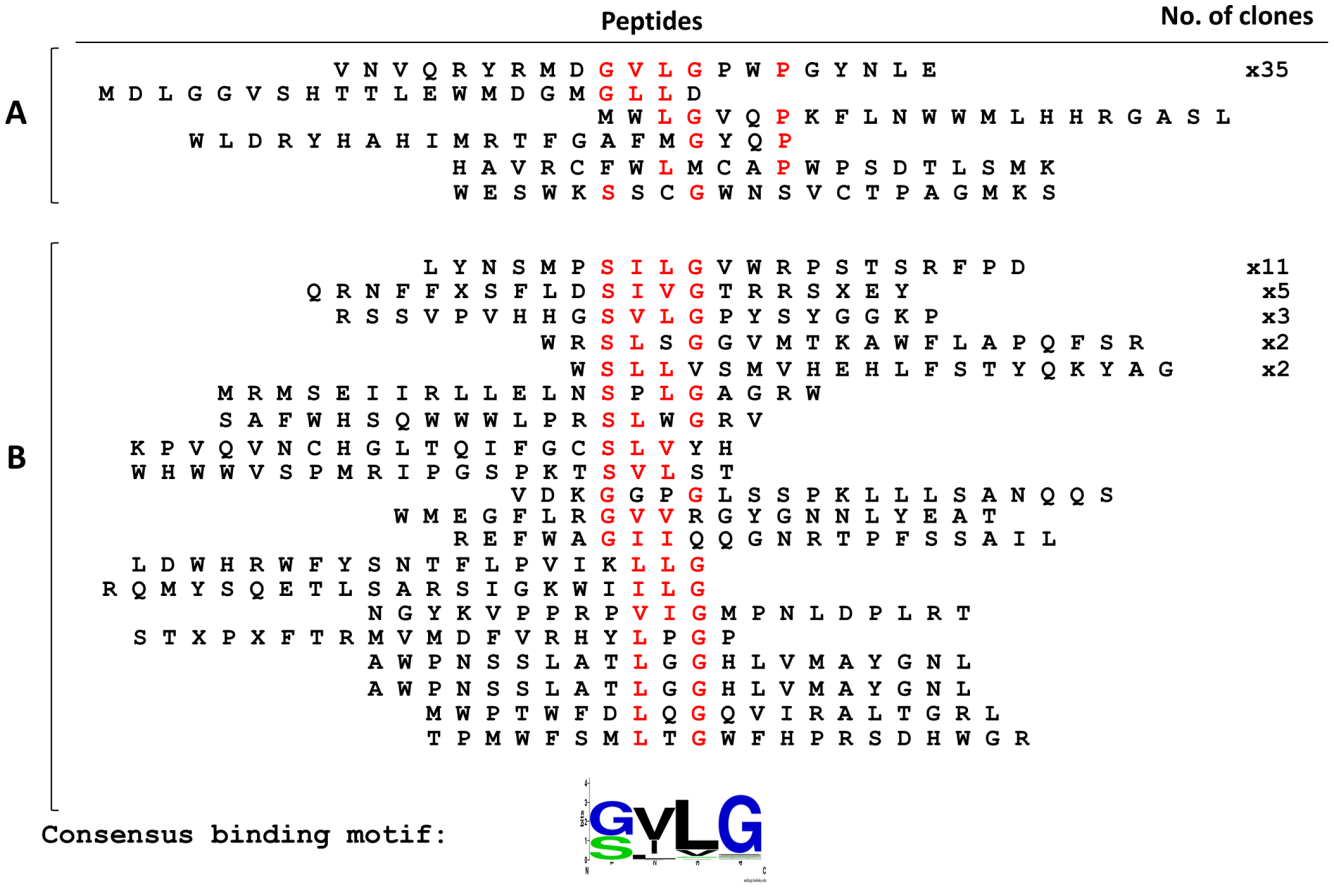
Phage-peptide library panning of *St*PTB proteins. (A) phage-peptides specifically enriched for binding with *St*PTB6 protein after four rounds of panning (B) phage-peptides specifically enriched for binding with *St*PTB1 protein after four rounds of panning. A consensus binding motif ‘[S/G][V/I][L/V]G’ was identified for *St*PTB protein from these peptide sequence using Weblogo [23].

The interaction between mammalian PTB and Raver1 protein is well studied and it has been shown that PTB recognize a consensus binding motif [S/G][I/L]LGxxP present on the C-terminus of Raver1 [8], [21] as well as Raver2 protein [7]. In order to ascertain if the *St*PTB proteins also recognize a specific binding motif, we used a random 21-mer phage peptide library to screen against both *St*PTB1 and *St*PTB6 as described in Materials and Methods. The phage display technique is widely used to identify peptide ligands, probe structure-function relationships in proteins and map protein-protein interaction surfaces [22]. Typically, phage-display screening yields peptides that contain linear sequence/motifs that have the potential to interact with the target molecule. Although the probability of finding the exact sequence of a binding partner is rather low, consensus analysis facilitates the identification of natural candidates from the database. Thus, after four rounds of panning, phages were obtained that were specifically enriched for binding with *St*PTB1 and *St*PTB6. One hundred clones were randomly selected, phagemids purified and checked for binding specificity against the respective target proteins in a phage-ELISA. Approximately 40 clones reacted positive for each protein and the derived peptide sequences are shown in Fig. 4. A single dominant peptide sequence, VNVQRYRMD**GVLG**PW**P**GYNLE (35 representations) recognized *St*PTB6 (Fig. 4A, top line) and the dominant sequence recognizing *St*PTB1 (11 representations) had the sequence LYNSMP**SILG**VWRPSTSRFPD (Fig. 4B, top line). From an analysis of all peptides recognizing both *St*PTB1 and −6 (Fig. 4), a consensus binding peptide motif with the sequence [S/G][V/I][L/V]G was identified by WebLogo analysis [23]. Interestingly, in the case of *St*PTB6, the recognition peptide contained not only the consensus sequence but also harbored the sequence ‘GVLGxxP’ that is similar to the mammalian PTB binding motif [S/G][I/L]LGxxP present on Raver1 protein. However, a search of the plant database did not reveal any Raver-like protein. Intriguingly, an amino acid sequence scan of *St*Nova1 revealed the sequence GIGGRYP located in the linker region between KH2 and KH3 domains that is similar to the GVLGPWP peptide identified in panning against *St*PTB6. It is worth noting that human Nova protein does not contain the[S/G][I/L]LGxxP like motif and, from the crystal structure of the Nova-RNA complex [24], it is inferred that the proline-alanine-glycine rich linker sequence between the KH2 and KH3 domains represents the protein-protein interaction surface.

### Determination of *St*PTB and *St*Nova1 Protein Interaction Interface via DIPP

**Figure 5:**
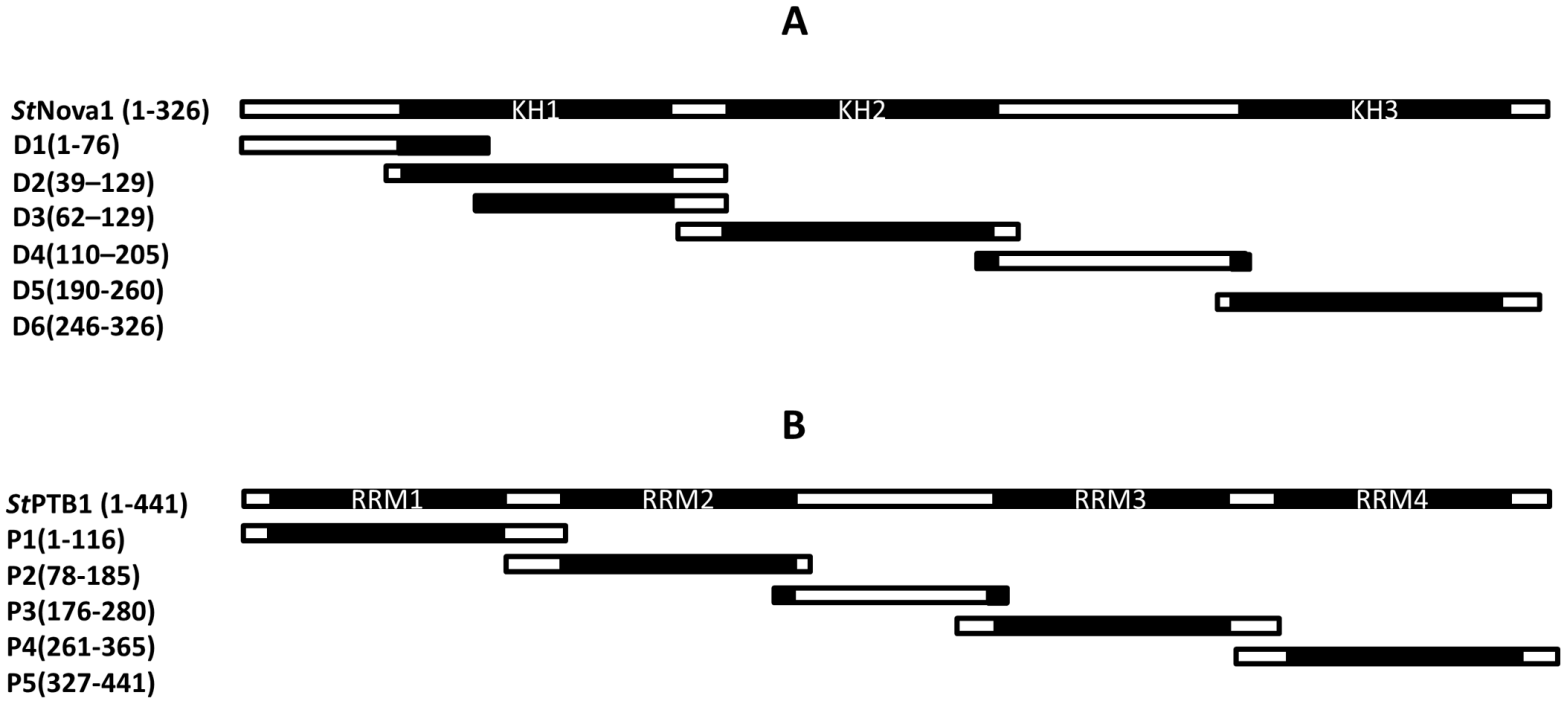
Schematic diagram of *St*Nova1 (A) and *St*PTB1 proteins (B) showing the arrangement of their respective KH domains and RRMs and their overlapping regions used to construct domain phage library.

**Figure 6:**
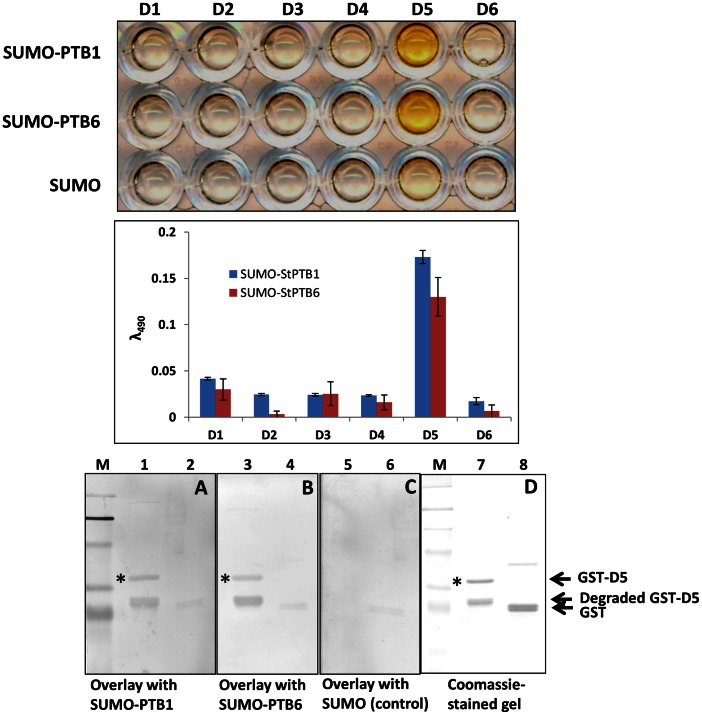
Mapping protein-protein interaction site on *St*Nova1 protein. (*Top panel)* Phage ELISA using *St*Nova1 single domain phage clones (D1 to D6) against SUMO-*St*PTB1, SUMO-*St*PTB6 and SUMO control. (*Middle panel*) Bar diagram showing quantification of phage binding. (*Bottom panel*) **A.** SUMO-*St*PTB1 protein overlay demonstrating its binding with GST-D5 region (lane 1, indicated with *), GST alone as a control shows no binding (lane 2) **B.** SUMO-*St*PTB6 protein overlay demonstrating its binding with GST-D5 region (lane 3, indicated with *), GST alone as a control shows no binding (lane 4) **C.** SUMO protein (as a control) overlay does not bind to either GST-D5 (lane 5) or GST alone (lane 6) **D.** Coomassie stained SDS PAGE showing protein inputs, GST-D5 (lane 7, indicated with *) and GST (lane 8) used for blotting PVDF membrane for protein overlay experiments.

**Figure 7:**
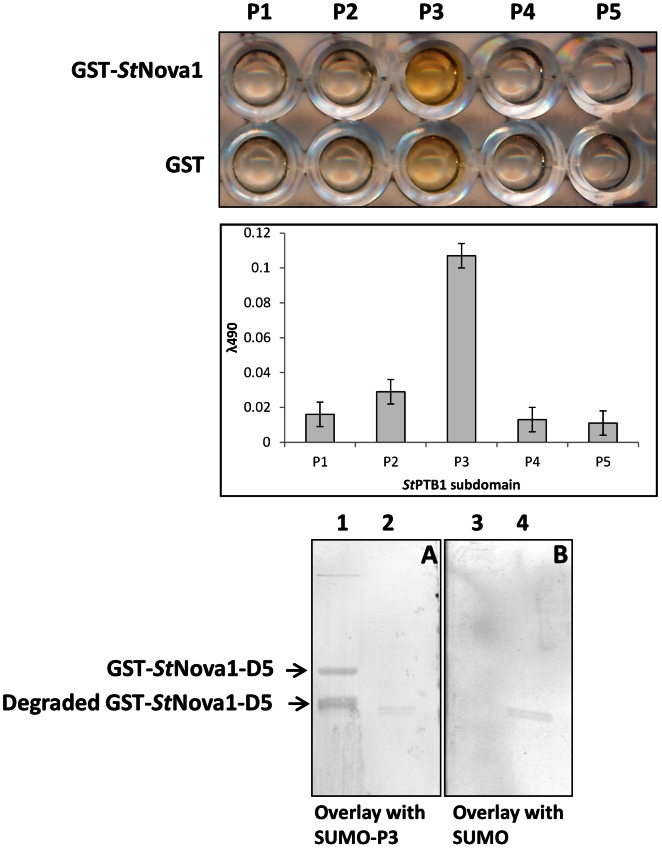
Mapping protein-protein interaction site on *St*PTB1. (*Top*) Phage ELISA using *St*PTB1 single domain phage clones (P1 to P5) against GST-*St*Nova1 protein and GST control (*Middle*) Bar diagram showing quantification of phage binding (*Bottom*) **A.** SUMO-P3 protein overlay demonstrating binding to GST-D5 region (lane1), GST alone as a control shows no binding (lane2) **B.** SUMO protein (control) overlay shows no binding to either GST-D5 (lane 3) or GST alone (lane 4).

To further delineate the specific sites of interaction between *St*PTB1/*St*PTB6 and *St*Nova1, we used a modified novel phage display technique termed DIPP. As described in Materials and Methods, *St*Nova1 was divided into six different overlapping domains D1–D6 (Fig. 5), cloned into phagemid vector and homogeneous phage particles prepared for each domain. SUMO-*St*PTB1 and SUMO-*St*PTB6 (and SUMO control) were immobilized on an ELISA plate and panned against a “domain phage library” prepared by mixing equal concentrations of individual phage subdomains. Additionally, phage-ELISA was performed with the individual domains. In the experiment with individual domains it is evident that only D5 (residues 190–260 in the linker region between KH2 and KH3) is capable of binding to both the PTB proteins (Fig. 6, top and middle panels). Further confirmation of the specificity of this interaction was obtained by protein overlay assays. Towards this end, the D5 region of *St*Nova1 was expressed as GST fusion and purified GST-D5 and GST (control) were blotted onto a PVDF membrane. The membrane was then overlaid with SUMO-PTB1/PTB6 protein and SUMO protein (control) followed by detection with anti-SUMO antibody as described in Materials and Methods. *St*PTB1 and *St*PTB6 were able to specifically bind with D5 region of *St*Nova1 (Fig. 6, bottom panel A and B). In a reciprocal experiment, five individual phage domains (P1–P5) and a domain library were prepared for the *St*PTB1 protein (Fig. 5B) and experiments performed against immobilized GST-*St*Nova1. With the individual subdomains, P3 (residues 176–280 in the linker region between RRM2 and RRM3) displayed the strongest specific binding (Fig. 7 top and middle panel). In a protein overlay assay, GST-D5 and GST protein were blotted onto membrane and incubated with either SUMO-P3 or SUMO protein. SUMO-P3 bound to the GST-D5 of *St*Nova1 (Fig. 7, bottom panel A, lane 1) but not to GST (Fig. 7, bottom panel A, lane 2). No binding was observed with SUMO alone (Fig. 7, bottom panel B, lanes 3 and 4). This experiment affirmed the specific interaction between the P3 region of *St*PTB1 and D5 of *St*Nova1.

In parallel experiments using subdomain phage libraries, after two rounds of panning, 20 randomly selected colonies were sequenced. D5 in *St*Nova1 was exclusively represented as the interaction partner for both *St*PTBs (Table 1) and P3 in *St*PTB1 as the predominant interaction partner for *St*Nova1 (Table 1). These results clearly demonstrate the specificity of interaction between linker regions of both the proteins and also verify that the respective RNA-binding domains in the two proteins are not involved in the interaction.

**Table 1:** Domain interaction phage panning: Series of *St*Nova1 and *St*PTB1 overlapping regions were cloned into phagemid vector and domain phage library was constructed for each proteins.

*St*Nova1 sub-domains	Frequency		
	*St*PTB1	*St*PTB6	
D1	0		0
D2	0		0
D3	0		0
D4	0		0
D5	20		20
D6	0		0
***St*** **PTB1 sub-domains**	**Frequency ** ***St*** **Nova1**		
P1	0		
P2	2		
P3	18		
P4	0		
P5	0		

The *St*PTB proteins were screened against *St*Nova1 domain phage library and *St*Nova1 was screened against *St*PTB1 domain phage library. After four rounds of panning 20 randomly selected clones were DNA sequenced. Table1 shows the frequency of peptide selectively binding to *St*PTB1 and *St*Nova1 proteins.

### Identifying the Binding Site within *St*Nova1 Linker (D5) Region

**Figure 8:**
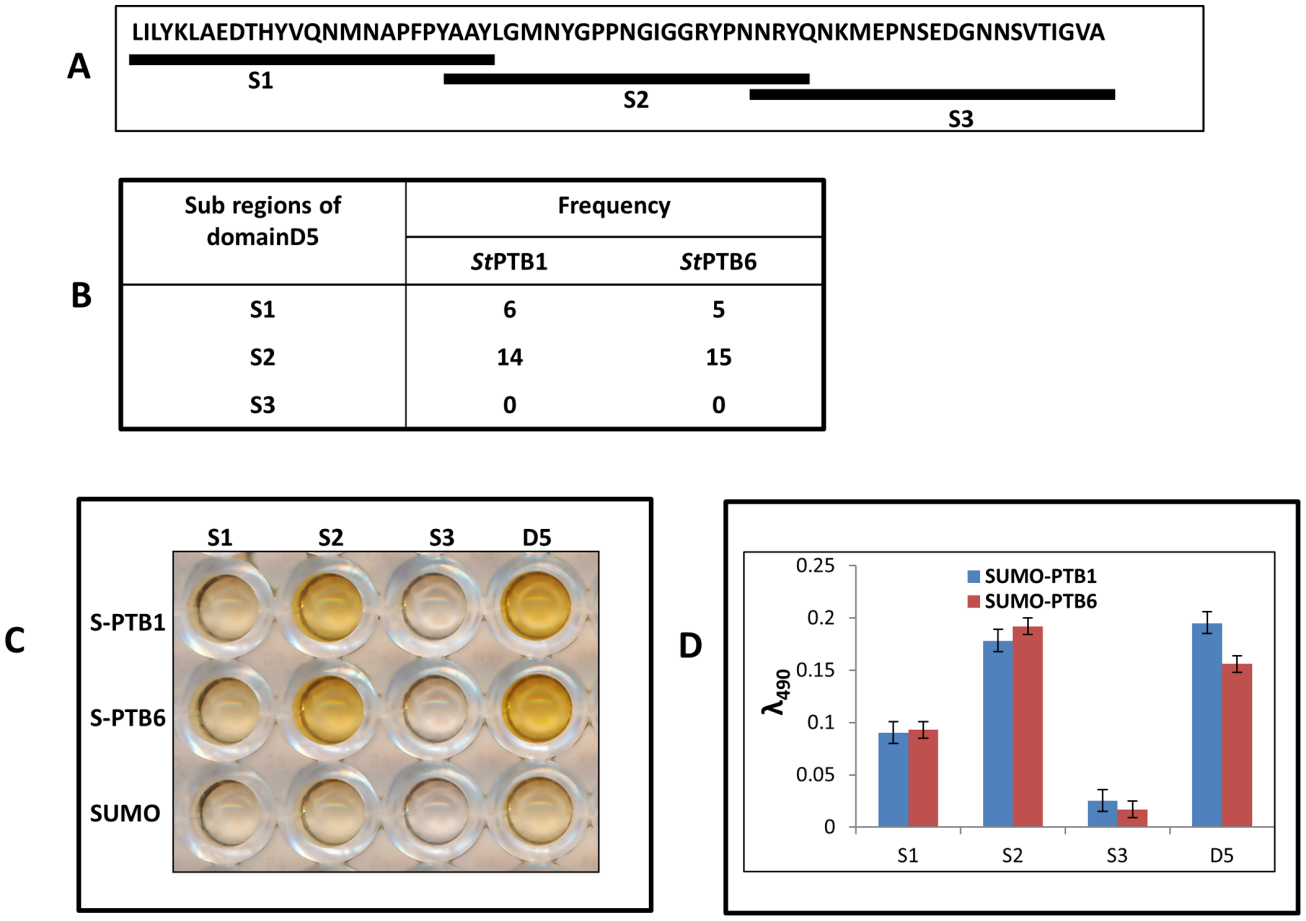
Determination of specific binding subdomain within *St*Nova1 D5. (**A**) Schematic showing the subdivision of D5 domain into three overlapping regions S1, S2 and S3 (**B**) Determination of specific binding region within D5 domain of *St*Nova1 by phage ELISA using phage clones of sub-domains S1, S2, S3 and domain D5 (**C**) Phage ELISA using phage clones of Domain D5 and sub-domains S1, S2, S3 against SUMO-*St*PTB1, SUMO-*St*PTB6 and SUMO control (**D**) Bar diagram showing quantification of phage binding.

**Figure 9:**
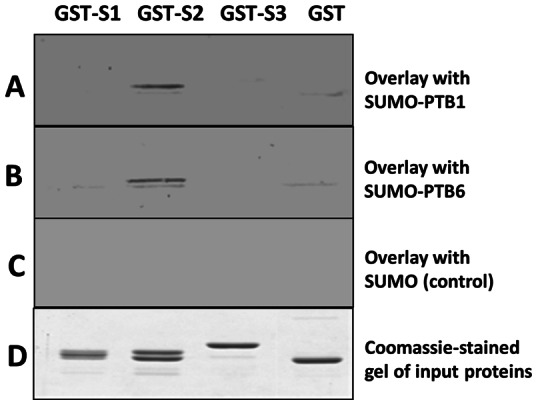
Binding of D5 sub-domains S1, S2 and S3 to *St*PTB proteins by protein overlay. GST-fused S1, S2 and S3 domains separated on SDS-PAGE were blotted on to PVDF membrane and probed with (**A**) SUMO-*St*PTB1 protein (**B**) SUMO-*St*PTB6 protein and (**C**) SUMO protein. Specific binding to the S2 domain is seen with the SUMO-PTB proteins (A and B) but not with SUMO (**C**). (**D**) Coomassie stained SDS PAGE of pure proteins. Binding was detected using anti-SUMO primary antibody, alkaline-phosphatase (AP) labeled secondary antibody and the AP substrate kit for color development as described in Materials & Methods.

To narrow down the binding sequence within D5, the linker region was further subdivided into three overlapping subdomains S1, S2 and S3 (Fig.8A) and a secondary phage domain library was constructed using these regions. After two rounds of panning against *St*PTB proteins, twenty randomly selected colonies were DNA sequenced. In the case of *St*PTB1, out of twenty colonies, fourteen colonies represented S2 and six colonies were for S1, whereas with *St*PTB6, 15 colonies represented S2 and five colonies were for S1 (Fig. 8B). Additionally, phage-ELISA performed with homogeneous phage particles of the individual sub-domains showed that the S2 subdomain has the strongest binding affinity towards *St*PTB proteins that is comparable to the binding of the entire D5 domain (Fig. 8C). In comparison, subdomain S3 displays no affinity and subdomain S1 shows weak binding. Further validation of these interactions came from protein overlays assays (Fig. 9) in which the strongest binding by the PTBs again was observed against the GST fused S2 subdomain. No interaction was observed with S1 and S3.

**Figure 10:**
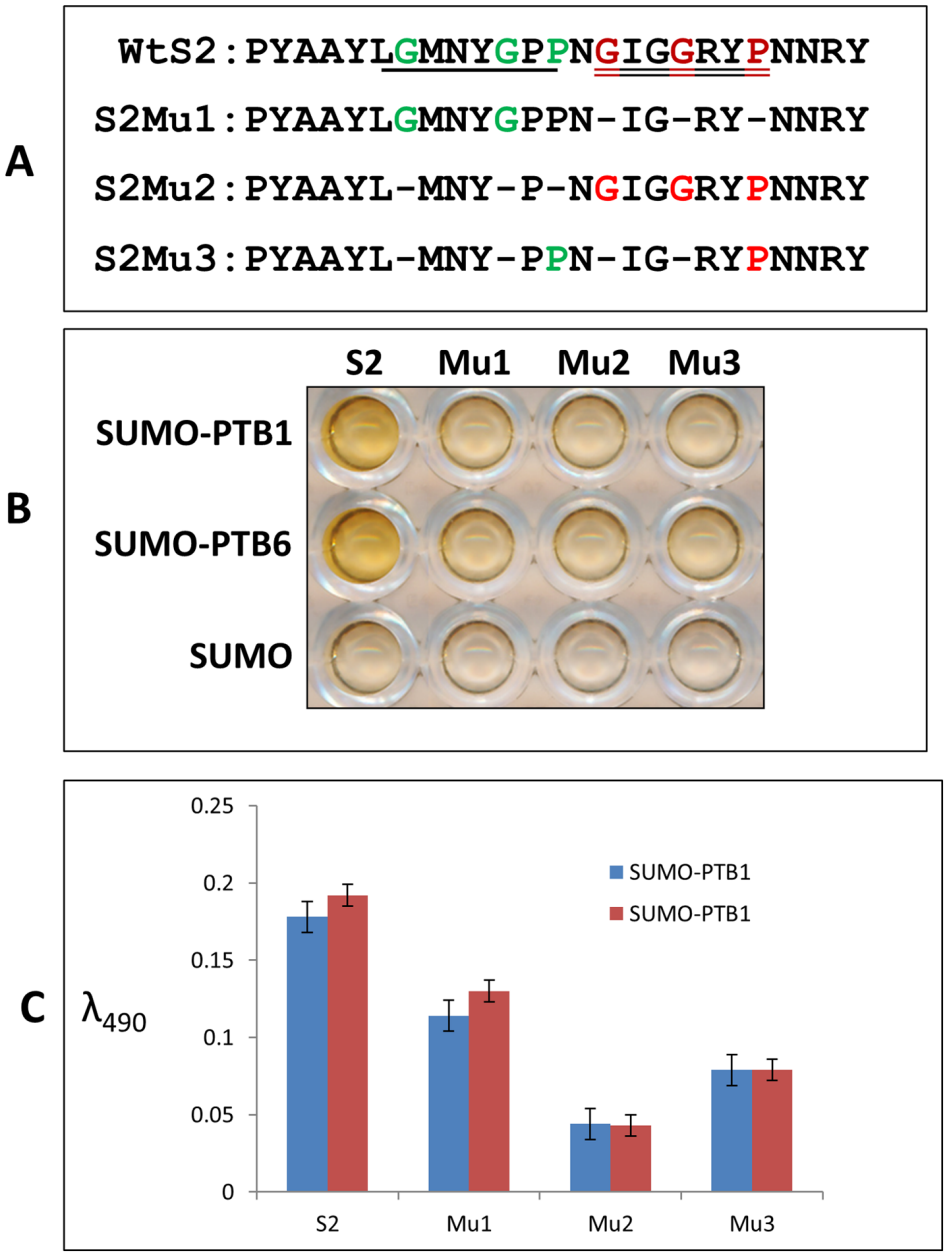
Mutational analysis to show that the glycine and proline rich motif of S2 region of *St*Nova1 is involved in binding with *St*PTB proteins. (**A**) Peptide sequence for S2 and deletion mutant S2Mu1, S2Mu2 and S2Mu3. The motifs ‘GxxxGxP’ and ‘GxxGxxP’ in the S2 peptide are underlined. (**B**) Phage ELISA showing that the deletion mutants of S2 have less affinity towards *St*PTB proteins compared to S2. (**C**) Bar diagram showing quantification of phage binding.

The combination of DIPP and phage display screening thus suggest that the D5 region of *St*Nova1 and specifically the S2 subdomain within may be most important for binding with *St*PTB proteins. Interestingly, contained within the S2 subdomain is the glycine/proline-rich sequence **G** MNY**G**P**P**N**G** I**GG**RY**P** (Fig. 10A). This stretch of sequence has two motifs GxxxGxP (underlined) and GxxGxxP (double underlined) that are similar to the motif present in the peptide VNVQRYRMDGVLGPWPGYNLE isolated in phage-panning experiments against *St*PTB6. Given the important role of Gly and Pro residues in protein-protein interactions [25] we checked the involvement of some of these residues in the S2 subdomain through three deletion mutants (Fig. 10A). In mutants S2Mu1 and S2Mu2, the deletions targeted specific Gly and Pro residues within the GxxGxxP and GxxxGxP sequences respectively, whereas in S2Mu3, only the specific Gly residues in the entire S2 subdomain were deleted. The deletion mutants were cloned into phagemid vector and single clone phage particles were generated. Phage ELISA was performed with each of the homogeneous population of phages displaying the mutant subdomains against SUMO tagged *St*PTB1, *St*PTB6 and SUMO protein as control (Fig. 10B). In comparison with the wild-type S2, a reduction in binding affinity was observed for all three mutants in the order Mu2< Mu3< Mu1.

## Discussion

In RNA-binding proteins, the RRM domain is essential for both recognition and binding to specific target RNA molecules. One of the better characterized RNA-binding proteins in mammals, the PTB protein, is involved in numerous aspects of RNA metabolism including post-transcriptional processing and other distinct functions: mobility, stability, translation, and AS [2]. A newly discovered aspect of plant PTBs is their unique role as chaperones that transport RNA complexes [13]. Particularly well-studied is the role of mammalian PTB-proteins in AS. To a large extent, it is this biological context that has driven the structure and function analysis of PTB proteins and delineated the structural preferences of the RRMs in interactions with RNA. Simultaneously, structural studies have also underscored the mobility and dynamic nature of the linker regions in correctly orienting the RRMs towards specific substrates [26], [27], [28], [29].

It has become increasingly evident, however, that in addition to direct RNA interactions, the function of PTB proteins can also be fine-tuned by interactions with other proteins. Thus, in studies on the splicing repressor domain in mammalian PTB protein, regions outside of RRM2 and proximal to the C-terminal linker were demonstrated to serve as sites for recruitment of other proteins that were independent of the RNA-binding activity [30]. On the other hand, in the case of the splicing repression of α-tropomyosin mediated by interactions of PTB with the protein Raver1, crystallographic studies show that the peptide motif [S/G][I/L]LGxxP in Raver1 interacts specifically with a hydrophobic groove in RRM2 but not with the linker following it [8], [21]. The Raver1 protein contains four such motifs referred to as PTB-Raver1 interacting motifs (PRIs) that are essential for its function [8] and are also conserved in a related protein of unknown function, Raver2 [7]. Interestingly, however, in the yeast 2-hybrid analysis of both full-length and truncated constructs of Nova1 and Nova2 that interact with brain-enriched PTB (br-PTB), the spacer domain of Nova-2 (amino acids 230–407) was clearly established as the protein-protein interaction site that was sufficient and distinct from the RNA-binding sites [5], [7], [10].

In the present work we have validated the interaction between two RNA-binding proteins in potato, *St*PTB1/*St*PTB6 and *St*Nova1, through multiple techniques. The significant sequence homology of these plant proteins to their mammalian counterparts and the conserved domain architecture, which are also suggestive of conserved molecular mechanisms, is indeed revealed in our studies. We demonstrate that it is the ∼65 amino-acid linker region (D5) between the KH2 and KH3 domains of *St*Nova1 that specifically binds to the *St*PTBs (Fig. 6). This region is analogous to the much longer spacer domain of mammalian Nova-2 comprising ∼180 amino acids that is also sufficient for binding to brPTB [5]. More importantly, within the linker region of *St*Nova1, the binding site can be narrowed further to the peptide sequence AAYLGMNYGPPNGIGGRYPNNRYQ in subdomain S2 (Fig. 8). Reciprocally, it is the linker region (P3) comprising amino acid residues 176–280 of *St*PTB1 (Fig. 5) that binds to *St*Nova1 (Fig. 7, top panel) specifically in the D5 region (Fig. 7, bottom panel). Thus, the linker regions in both proteins, *St*PTB1/*St*PTB6 and *St*Nova1, constitute the “hotspots” of the protein-protein interaction interface while leaving their respective RRM domains free to simultaneously bind RNA and participate in other functions.

Hotspots are defined as essential amino acids that constitute a small subset of interface residues that are necessary and sufficient for binding between two proteins [31], [32]. In addition to predictive computational methods and alanine scanning mutagenesis to locate these sites, screening with a population of random peptides, such as those displayed on the surface of phage, has proven to be a powerful method for identifying “consensus” sequence motifs that mimic protein–protein interaction surfaces [22]. In many instances, peptide ligands isolated from phage-peptide libraries show conservation of such motifs with native interacting proteins, facilitating the identification of natural candidate proteins [33], [34], [35]. Along these lines, it is interesting that screening with a random, untrained phage-peptide library yields a single dominant peptide, VNVQRYRMDGVLGPWPGYNLE, that binds to *St*PTB6 (Fig. 4). Intriguingly, this peptide contains the sequence ‘GVLGPWP’ which is strikingly similar to the peptide motif ‘[S/G][I/L]LGxxP’ in Raver1 protein that is essential for interaction with mammalian PTB. A similar motif, GIGGRYP, occurs in the sequence AAYLGMNYGPPNGIGGRYPNNRYQ in the S2 region of *St*Nova1 and specifically interacts with *St*PTB (Fig. 8). The significant decrease in binding efficiency that occurs upon deletion of the Gly and Pro residues within the GIGGRYP and GMNYGPP sequences, (Fig. 10) illustrates the critical dependence of just a few amino acids that contribute to the energetics of interactions within a hotspot region. It is also interesting to note that the sequence alignment of the spacer region between the KH2 and KH3 domains of Nova-like proteins from other plant species shows high similarity (Fig. S1). This suggests that, in general, the Gly-rich sequence within the spacer region of plant Nova-like proteins may also serve as a site for protein-protein interaction. Perhaps, more importantly, the conservation of a protein interaction motif, GxxGxxP in two unrelated and non-homologous proteins, mammalian Raver1 and plant *St*Nova1, is illustrative of the plasticity of residues in the hotspot to adapt to different structural contexts imposed by dissimilar sequences, while retaining the ability to perform similar functions of molecular recognition [25], [36]. However, in the case of human Nova the linker region does not contain any similar motifs and potential interactions with other proteins may depend on determinants specified by the sequence. It is also worth noting that the amino acid sequence of the linker region (P3) between RRM2 and RRM3 in other plant PTB-like proteins is highly conserved (Fig. S2), suggesting also a possible conservation of function as a protein interaction interface in these proteins.

In conclusion, our studies indicate that the interaction between the RNA-binding proteins *St*PTB1/6 and *St*Nova1 is mediated through linker regions that are distinctly separated from the RRMs. While the molecular details of this interaction and the specific involvement of the RRMs will have to await atomic level resolution of the structure with and without RNA, one can gain some understanding by drawing upon the extensive structural investigations of mammalian PTBs in complex with RNA and proteins and their mechanistic implications in AS [2], [27], [29]. Such studies provide strong evidence for the formation of ternary complexes driven by cooperative interactions involving all the RRMs and the linker regions. Therefore, given the multiple RRMs and separate (or overlapping) protein interaction surfaces in *St*PTB and *St*Nova1, it is entirely reasonable that they can provide the scaffolding for macromolecular assembly of specific proteins and RNA for multidimensional regulation of various processes. Included among these functions is the potential ability to facilitate long-distance RNA transport. To this extent, identification of specific protein-binding regions outside of the RRMs suggests independent functional interaction of these domains within the PTB/Nova complex in plants. Support for this comes from the characterization of a large ribonucleoprotein complex in pumpkin that binds phloem-mobile RNAs. Mediated by the 50 kDa PTB protein, RBP50, this complex contains six RNAs and sixteen other proteins [13]. Although no Nova-like protein was identified in this complex, a KH-domain protein very similar in sequence to *St*Nova1 was identified in the phloem sap of pumpkin [37], suggesting a role for Nova proteins in long-distance transport of mRNAs. *St*PTB1 and −6 are the potato orthologs of the pumpkin RBP50 and have been implicated in interactions with full-length phloem-mobile mRNAs that function in plant development [38]. One of these, *StBEL5*, is a phloem-mobile RNA that is involved in regulating tuber formation [39]. Coupled to the fact that both *St*PTB1 and −6 can also bind to CU-rich sequences in the 3′ untranslated region of *StBEL5*, a plausible argument can be made for the existence of a RBP50-like complex in potato similarly involved in long-distance mobile RNA transport. We are currently pursuing studies to establish the identity of this complex and its components.

## Supporting Information

Figure S1 **Sequence alignment of linker region between KH2 and KH3 domain of plant Nova-like proteins.** Sequence identification number appears in brackets following the species name.
(DOCX)

Figure S2 **Sequence alignment of linker region between RRM2 and RRM3 domain of plant PTB like proteins.** Sequence identification number appears in brackets following the species name.
(DOC)

Table S1 **List of forward and reverse primers.**
(DOC)
