# Supplementary material for: Mapping and Characterization of the Interaction Interface between Two Polypyrimidine-Tract Binding Proteins and a Nova-Type Protein of Solanum tuberosum
Source: PLoS One. 2013 May 24;8(5):e64783. doi: 10.1371/journal.pone.0064783 (PMC3663837; doi:10.1371/journal.pone.0064783)
Supplement: Figure S1 — Sequence alignment of linker region between KH2 and KH3 domain of plant Nova-like proteins. Sequence identification number appears in brackets following the species name. (DOCX) [file pone.0064783.s001.docx]

**Figure S1**

***Populus trichocarpa(*ABK95306.1)** LSKLTDDTHYLQNMHAPLSYA---------------------AAYN-STNHGLNG-AGVKFQHNK----------EDRTNS 48

***Ricinus communis(*XP_002532622.1)** LSKLYDDPHYVQTMHAPFSYA---------------------VAYN-SMNYGANG-AGGKFQNNK----------EDRTNS 48

***Vitis vinifera*(XP_002264331.2)**  LSKLTEDPHYTQFMNAPFSYA---------------------AAYN-SMNYGPNG-AGGKFQNNK----------EDRSNS 48

***Glycine max*(XP_003536289.1)**  VSKLSEDPHYAQSMNSPFSYPGVYFSGYQGVPYTYVLPSVAPPAYN-AVNYRPNGAAGGKLQNSK----------EERSNS 70

***Arabidopsis thaliana(*NP_196063.1)**LAKLTEDDHYSQNVHSPYSYA---------------------AGYN-SVNYAPNG-SGGKYQNHK----------EEAST- 47

***Oryza sativa (dbj|BAD22893.1)***  LSKLSEDVHYPPNLSSPFPYAGLGFPSYPGVPVGYMIP---QVPYNNAVNYGPN-GYGGRYQNNKPSTPMRSPANNDAQDS 77

***Zea mays (*ACF84977.1)**  LKKLSEDVHYPANLSSPFPYAGLTFPSYPGVPVGYMIP---QVPYNNAVNYGPNNGYGGRYQNNKPSTPMRSPASNEAQES 78

***Solanum tuberosum***  LYKLAEDTHYVQNMNAPFPYA----------------------AYL-GMNYGPPNGIGGRYPNNRYQNKME-PNSEDGNNS 57

: ** :* ** : :* .*. * . *: * : : : ::
