# Supplementary material for: Mapping and Characterization of the Interaction Interface between Two Polypyrimidine-Tract Binding Proteins and a Nova-Type Protein of Solanum tuberosum
Source: PLoS One. 2013 May 24;8(5):e64783. doi: 10.1371/journal.pone.0064783 (PMC3663837; doi:10.1371/journal.pone.0064783)
Supplement: Figure S2 — Sequence alignment of linker region between RRM2 and RRM3 domain of plant PTB like proteins. Sequence identification number appears in brackets following the species name. (DOC) [file pone.0064783.s002.doc]

**Figure S2.**

*Cicer arietinum*(emb|CAD70621.1) NLDELQVNYNNDRSRDYTNPNLPTEQKGRPSH-SGYGDTG-MHGVQGSGARPGGFSQMTN 58

*Glycine max*(XP_003536416.1) NLDELQVNYNNDRSRDFTNPNLPTEQKGRPSQ-PGYGDAGNMYAAQGSGARAVGFPQMAN 59

*Arabidopsis thaliana*(NP_001077673.1) NLEELQVNYNNDRSRDYTNPNLPAEQKGRSSH-PCYGDTG------------VAYPQMAN 47

*Solanum tuberosum* (gb|AEG89703.1|) NLDELQVNYNNERSRDYTNPNLPSEQKGKSSQ-QGYGD---MYSFQGSGAHPGGFPQMGN 56

*Solanum tuberosum* (gb|AEG89704.1|) NLDELQVSYNNERPRDFTNPNLPSEPKGKSPQ-QGYGDAGAMYPWQGSGPRGVGFPQMGN 59

*Populus trichocarpa*(|XP_002302167.1) NLDELQVNYNNDRSRDFTNPNLPSEQKGRSSQ-------------VCTGLLTIYHPLMPN 47

*Cucurbita maxima*(gb|ACI43571.1|) NLDELQVNYNNERSRDFTNPSLPSEPKGRPSQQPGYGDAGGMYALQASGAGPVGFPQMAN 60

*Cucumis sativus*(|XP_004138370.1|) NLDELQVNYNNERSRDFTNPSLPSEPKGRSSQ-PGYGDTGGMYPLQPSGARPVGFSQMAN 59

*Vitis vinifera*(|XP_003632518.1|) NLTELQVNYNNERSRDFTNPSLPSEQKGRSSQ-SGYGDGGGMYALQPPGARPVAFPQMGN 59

*Ricinus communis*(|XP_002524303.1|) NLDELQVNYNNDRSRDFTNPHLPAEQKGRSSQ-AGYGDAG------------VAYPQMAN 47

*Oryza sativa*(|NP_001055652.1|) NLSELQVHYNNDRSRDFTNPSLPTEQRPRASQ-QGYPDPGGLYAFQQPG---ASYAQMGR 56

*Zea mays* (|NP_001169470.1|) NLSELQVHYNNDRSRDFTNPSLPTEQRPRASQ-QGYLDPANLYAFQQAG---ASYAQMGR 56

** **** ***:*.**:*** **:* : :..: .. * .

*Cicer arietinum*(emb|CAD70621.1) AAAIEAAFGGDLPPGITGTNDRCTVLVANLNPDRIDEDKLFNLFSIYGN 107

*Glycine max*(XP_003536416.1) AAAIAAAFGGGLPPGITGTNDRCTVLVSNLNPDRIDEDKLFNLFSIYGN 108

*Arabidopsis thaliana*(NP_001077673.1) TSAIAAAFGGGLPPGITGTNDRCTVLVSNLNADSIDEDKLFNLFSLYGN 96

*Solanum tuberosum* (gb|AEG89703.1|) AEAIAAAFAGGLPPGISGTNDRCTILVSNLNSDRINEDKLFNLCSLYGN 105

*Solanum tuberosum* (gb|AEG89704.1|) AAAIATAFPSGLPPGISGTNDRCTIIVSNLNPDRIDEDKLFNLFSIYGN 108

*Populus trichocarpa*(|XP_002302167.1) AAAIAAAFGGGLPPGISGTNDRCTVLASNLNPDRIDEDKLFNLFSLYGN 96

*Cucurbita maxima*(gb|ACI43571.1|) AAAVAAAFGGGLPPGVSGTNDRCTVLVSNLNPDRIDEDKLFNLFSIYGN 109

*Cucumis sativus*(|XP_004138370.1|) AAAVAAAFGGGLPPGVSGTNDRCTVLVSNLNPDRIDEDKLFNLFSIYGN 108

*Vitis vinifera*(|XP_003632518.1|) ATAIAAAFGGGLPPGISGTNDRCTVLVSNLNPDEIDEDKLFNLFSLYGN 108

*Ricinus communis*(|XP_002524303.1|) AAAIAAAFGGGLPPGISGTNDRCTVLVSNLNPDKIDEDKLFNLFSLYGN 96

*Oryza sativa*(|NP_001055652.1|) AAMITAAFGGTLPPGVTGTNERCTLIVSNLNTDKINEDKLFNLFSLYGN 105

*Zea mays* (|NP_001169470.1|) VAMIAAAFGGTLPHGVTGTNERCTLIVSNLNTDKIDEDKLFNLFSLYGN 105

. : :** . ** *::***:***::.:***.* *:******* *:***
