# Supplementary material for: Mapping and Characterization of the Interaction Interface between Two Polypyrimidine-Tract Binding Proteins and a Nova-Type Protein of Solanum tuberosum
Source: PLoS One. 2013 May 24;8(5):e64783. doi: 10.1371/journal.pone.0064783 (PMC3663837; doi:10.1371/journal.pone.0064783)
Supplement: Table S1 — List of forward and reverse primers. (DOC) [file pone.0064783.s003.doc]

| **Gene** | **Vector** | **Forward Primer** | **Reverse Primer** |
| --- | --- | --- | --- |
| ***St*PTB1** | pESUMO | GGTGGTGGTGGTCTCAAGGTATGTCTGATCCTTCAAAGGTT | GGTGGTGGTGGTCTCTCTAGATCAGATGCTCTGTAACTGAGAA |
| ***St*PTB1** | pGEX4T-1 | TTCGGGTTGCGGCCAATGTCTGATCCTTCAAAGGTT | TGCGGCCCGGCGGCCACTATCAGATGCTCTGTAACTGAGAA |
| ***St*PTB6** | pESUMO | GGTGGTGGTGAAGACAAAGGTATGACTGAGCCGTCAAAAGTC | GGTGGTGGTTCTAGATCATCAGATGCTTTGCACCTGAG |
| ***St*PTB6** | pGEX4T-1 | TTCGGGTTGCGGCCAATGACTGAGCCGTCAAAAGTC | TGCGGCCCGGCGGCCACTAGATGCTTTGCACCTGAG |
| ***St*Nova1** | pGEX4T-1 | TTCGGGTTGCGGCCAATGGCTATGAAGGAGCAGC | TGCGGCCCGGCGGCCACTATCAACTCTCAGTAACTGTTGC |
| **Nova-D1** | pCANTAB 5E | GGTGGTGGTGGCCCAGCCGGCCATGGCTATGAAGGAGCAGC | GGTGGTGGTGCGGCCGCCTGAATTCTTGCCCCAGAAC |
| **Nova-D2** | pCANTAB 5E | GGTGGTGGTGGCCCAGCCGGCCCTGAGGGCTGGTTCAATTATTG | GGTGGTGGTGCGGCCGCTTTTGATCGCGGGCCAACAT |
| **Nova-D3** | pCANTAB 5E | GGTGGTGGTGGCCCAGCCGGCCTCGACAATCAGTGACTTTCA | GGTGGTGGTGCGGCCGCTTTTGATCGCGGGCCAACAT |
| **Nova-D4** | pCANTAB 5E | GGTGGTGGTGGCCCAGCCGGCCAATAAACTACTGGATGAGAGTTA | GGTGGTGGTGCGGCCGCTACATAATGAGTGTCTTCAGC |
| **Nova-D5** | pCANTAB 5E | GGTGGTGGTGGCCCAGCCGGCCATTGAATTGATTCTATATAAGTTG | GGTGGTGGTGCGGCCGCACGCTCATCAGCAACACCAA |
| **Nova-D6** | pCANTAB 5E | GGTGGTGGTGGCCCAGCCGGCCGAAGATGGGAACAATTCTGTCACT | GGTGGTGGTGCGGCCGCTCAACTCTCAGTAACTGTTGC |
| **PTB-P1** | pCANTAB 5E | GGTGGTGGTGGCCCAGCCGGCCATGTCTGATCCTTCAAAGGTT | GGTGGTGGTGCGGCCGCCACATCCACGGTTATAGGGT |
| **PTB-P2** | pCANTAB 5E | GGTGGTGGTGGCCCAGCCGGCCTCTCACCAAGAATTGACAACTA | GGTGGTGGTGCGGCCGCATTGTAGTTCACTTGCAATTCA |
| **PTB-P3** | pCANTAB 5E | GGTGGTGGTGGCCCAGCCGGCCAACCTAGATGAATTGCAAGTGA | GGTGGTGGTGCGGCCGCATTTCCATAAAGAGAGCACAG |
| **PTB-P4** | pCANTAB 5E | GGTGGTGGTGGCCCAGCCGGCCTTAAATTCAGATAGAATAAATGAG | GGTGGTGGTGCGGCCGCCATTTTGGTAGGGGAGCAGCA |
| **PTB-P5** | pCANTAB 5E | GGTGGTGGTGGCCCAGCCGGCCAAGTACCCCAATATTACTACAG | GGTGGTGGTGCGGCCGCGATGCTCTGTAACTGAGAAAAT |
| **Nova-S1** | pCANTAB 5E | CGGCCTTGATTCTATATAAGTTGGCTGAAGACACTCATTATGTACAGA  ACATGAATGCTCCATTTCCTTATGCAGCTTATGC | GGCCGCATAAGCTGCATAAGGAAATGGAGCATTCATGTTCT  GTACATAATGAGTGTCTTCAGCCAACTTATATAGAATCAAGGCCGGCT |
| **Nova-S2** | pCANTAB 5E | CGGCCCCTTATGCAGCTTATCTTGGAATGAACTATGGACCACCGAATG  GAATTGGAGGGAGATATCCAAATAACAGATATGC | GGCCGCATATCTGTTATTTGGATATCTCCCTCCAATTCCATTCG  GTGGTCCATAGTTCATTCCAAGATAAGCTGCATAAGGGGCCGGCT |
| **Nova-S3** | pCANTAB 5E | CGGCCCCAAATAACAGATATCAGAACAAGATGGAGCCTAATTCTGAAG  ATGGGAACAATTCTGTCACTATTGGTGTTGCTGC | GGCCGCAGCAACACCAATAGTGACAGAATTGTTCCCATCTTCA  GAATTAGGCTCCATCTTGTTCTGATATCTGTTATTTGGGGCCGGCT |
| **S2Mu1** | pCANTAB 5E | CGGCCCCTTATGCAGCTTATCTTGGAATGAACTATGGACCACCGAATA  TTGGAAGATATAATAACAGATATGC | GGCCGCATATCTGTTATTATATCTTCCAATATTCGGTGGTCCAT  AGTTCATTCCAAGATAAGCTGCATAAGGGGCCGGCT |
| **S2Mu2** | pCANTAB 5E | CGGCCCCTTATGCAGCTTATCTTATGAACTATCCAAATGGAATTGGAGG  GAGATATCCAAATAACAGATATGC | GGCCGCATATCTGTTATTTGGATATCTCCCTCCAATTCCATTTGGAT  AGTTCATAAGATAAGCTGCATAAGGGGCCGGCT |
| **S2Mu3** | pCANTAB 5E | CGGCCCCTTATGCAGCTTATCTTATGAACTATCCACCGAATATTAGATAT  CCAAATAACAGATATGC | GGCCGCATATCTGTTATTTGGATATCTAATATTCGGTGGATAGT  TCATAAGATAAGCTGCATAAGGGGCCGGCT |

**Table S1**:
